# Strong gene activation in plants with genome‐wide specificity using a new orthogonal CRISPR/Cas9‐based programmable transcriptional activator

**DOI:** 10.1111/pbi.13138

**Published:** 2019-05-23

**Authors:** Sara Selma, Joan Miquel Bernabé‐Orts, Marta Vazquez‐Vilar, Borja Diego‐Martin, Maria Ajenjo, Victor Garcia‐Carpintero, Antonio Granell, Diego Orzaez

**Affiliations:** ^1^ Instituto de Biología Molecular y Celular de Plantas (IBMCP) Consejo Superior de Investigaciones Científicas Universidad Politécnica de Valencia Valencia Spain

**Keywords:** CRISPR/Cas9, dCas9, scRNA, transcriptional activation, synthetic biology, GoldenBraid

Synthetic biology (SynBio) aims at rewiring plant metabolic and developmental programmes with orthogonal regulatory circuits. This endeavour requires new molecular tools able to interact with endogenous factors in a potent yet at the same time highly specific manner. A promising new class of SynBio tools that could play this function are the synthetic transcriptional activators based on CRISPR/Cas9 architecture, which combine autonomous transcriptional activation domains (TADs) capable of recruiting the cellular transcription machinery, with the easily customizable DNA‐binding activity of nuclease‐inactivated Cas9 protein (dCas9), creating so‐called programmable transcriptional activators (PTAs). Initial dCas9‐PTAs versions comprising single domain fusions to Cas9 showed only low/moderate activation rates in plants. A new generation PTAs developed for mammalian cells combine several TADs attached to a single dCas9 protein, reaching higher activation rates. Multi‐TAD display can be achieved following different strategies (see Figure 1b). The SunTag strategy (Tanenbaum *et al*., 2014) uses multiepitope tags to attach multiple TADs to the dCas9 protein, whereas SAM and scRNA strategies (Konermann *et al*., 2015; Mali *et al*., 2013) use RNA aptamers added to the gRNA scaffold as secondary anchoring sites for TADs. Recently, some multi‐TAD designs have been successfully applied to plants (Li *et al*., 2017; Lowder *et al*., 2018); however, a comprehensive multiparallel test for plant dCas9‐PTAs including multi‐TAD designs is still missing, leaving the door open for design improvements. Here, we show a systematic comparison of 43 SunTag, SAM and scRNA‐based TAD combinations tested for their ability to activate different promoters fused to a luciferase reporter.

**Figure 1:**
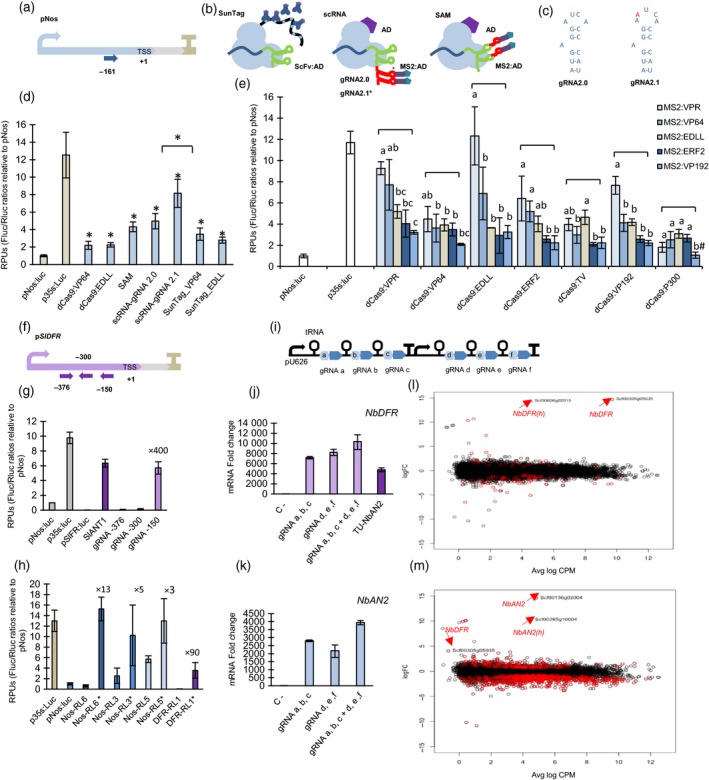
(a) Representation of *Nos* promoter with a gRNA target at position −161. (b) Schematic representation of SunTag, scRNA (gRNA2.0 or gRNA 2.1) and SAM strategies. Asterisk indicates the mutation position in gRNA2.1. (c) Representation of the MS2 aptamer loop in 2.0 and 2.1 variants. (d) Relative transcriptional activities (RTAs) obtained with different dCas9‐PTAs strategies. All SAM and scRNA strategies combine direct dCas9‐EDLL fusions with gRNA aptamers attached to MS2‐VP64. RTAs of p*N* *os* and p*35S* reporters measured in the same example are also included as a reference. Asterisks indicate Student's *t‐test* significant values (*P* < 0.05). (e) RTAs obtained upon activation with different combinations of TADs using scRNA‐gRNA 2.1 strategy, targeting the reporter pNos:Luc at position −161. ANOVA test was performed for each dCas9‐PTA group. Bars sharing the same letter are not significantly different according to Tukey's HSD test (*P* < 0.05). The # symbol indicates no statistical differences with the control sample. (f) Representation of the reporter pSlDFR:Luc with gRNAs targeted at positions −150, −300 and −376. (g) Reporter RTAs measured in leaves transiently transformed with p*S* *lDFR*:Luc alone or in combination with SlANT1 or dCasEV2.1 targeting positions −150, −300 and −376 of p*S* *lDFR*. (h) RTAs measured in transgenic lines containing stably transformed p*N* *os* and p*S* *lDFR*‐driven reporters. The p*N* *os* reporter lines (Nos‐RL) were activated by targeting the p*N* *os* promoter at positions −161 and −211. DFR‐RL5 line was activated by targeting the p*S* *lDFR* promoter at position −150. Asterisk labels indicate the presence of functional dCasEV2.1 activator as compared with nonactivated control samples. (i) Representation of the multiplexing strategy for dCasEV2.1 activation. (j) mRNA fold change at 4 dpi obtained by targeting the endogenous gene *NbDFR* with dCasEV2.1 using the multiplexing strategy. The depicted gRNAs target p*N* *bDFR* promoter at the positions: a (−88), b (−125), c (−217), e (−198) and f (−248). (k) mRNA fold change at 4 dpi obtained by targeting the endogenous *NbAN2* gene with dCasEV2.1 using a multiplexing gRNA strategy. The gRNAs a‐f were designed to target the promoter of *NbAN2* at the following positions: a (−103), b (−175), c (−196), d (−145), e (−198) and f: (−252). (l) Differential gene expression plot between the dCasEV2.1–*NbDFR* condition and control condition. (m) Differential gene expression plot between the dCasEV2.1–*NbAN2* condition and control condition. Bars represent average RTAs ± SD, *n* = 3 in all experiments.

A first round of comparisons among the different PTA dCas9 strategies was performed transiently in *Nicotiana benthamiana* leaves. PTA transactivation levels were assessed using the nopaline synthase promoter (p*Nos*) coupled to firefly luciferase (Fluc) reporter. A constitutive Renilla luciferase (Rluc) was used as internal reference driven by CaMV35s promoter. For initial comparisons, PTAs were targeted to the p*Nos* with a single gRNA annealing at position −161 relative to the transcriptional start site (TSS; Figure 1a). Previous analysis by Vazquez‐Vilar *et al*. (2016) showed only moderate transcriptional activation levels (maximum 3×) using VP64 or EDLL single fusions to Cas9. To assess multi‐TAD approaches, we analysed the same activation domains in a combinatorial mode using SAM, scRNA and SunTag designs (Figure 1b). For SunTag, VP64 and EDLL were fused to a GCN4‐ScFv antibody and tested separately. For scRNA and SAM, EDLL was attached to dCas9 and VP64 was fused to the Ms2 viral coat protein, which binds Ms2 RNA aptamers. RNA scaffolds in scRNA and SAM designs contained a second optimized aptamer next to the wild‐type one, as this double‐aptamer design (gRNA2.0) was earlier found to improve binding activity (Nowak *et al*., 2016). During the cloning of the gRNA2.0 scaffold employed in the scRNA design, a spontaneous mutation occurred consisting in the insertion of an adenine in the loop of the first aptamer (Figure 1c). Since this aptamer variant had not been studied earlier, we decided to include it in the comparison analysis (labelled as gRNA2.1). The experiment was completed with direct dCas9:VP64 and dCas9:EDLL fusions. Relative transcriptional activities (RTAs) in transient assays were expressed as relative transcription units (RPUs). RPUs are calculated as the Fluc/Rluc ratios measured in each sample, normalized with the Fluc/Rluc ratios produced by an unactivated p*Nos* promoter (Catalogued as GB1398, https://gbcloning.upv.es/) assayed in parallel. This procedure was earlier proposed as a standard measurement for the documentation of standard DNA parts (phytobricks; Vazquez‐vilar *et al*., 2017). In these conditions, the CaMV35S promoter‐based standard DNA part, catalogued as GB0164, consistently confers activity levels of 12 ± 2 RPUs and was included in all comparative experiments as an upper limit reference. Surprisingly, the highest transcriptional activation was achieved with scRNA‐gRNA2.1, reaching RTA levels close to CaMV35S (RTA = 8 ± 2 RPUs) and outperforming previously optimized scRNA‐gRNA2.0 strategy. In a further optimization step, we tested the scRNA2.1 design with new TAD combinations covering different types of autonomous TADs, namely two plant transcriptional factors (TFs; EDLL, ERF2), two viral TFs (VP64, VP192), a chromatin modifier (P300) and two combined domain fusions (VPR, TV; Chavez *et al*., 2015; Li *et al*., 2017). Among all combinations assayed, dCas9:EDLL‐MS2:VPR gave maximum p*Nos* activation up to RTA levels similar to the CaMV35S reference.

The results obtained with the pNos:Luc reporter prompted us to test the activity of dCas9:EDLL‐MS2:VPR/gRNA2.1 (now abbreviated as dCasEV2.1) on promoters with low basal activities, characteristic of strongly repressed genes. The promoter of the *SlDFR* gene (dihydroflavonol‐4‐reductase) from *Solanum lycopersicum* (p*SlDFR*, catalogued as GB1160) has very low basal expression levels (RTA < 0.04 RPUs), but it is strongly induced by the presence of ‘natural’ Myb TF (e.g. Sl*ANT1*). Thus, p*SlDFR* was used as model promoter to test the activation range of dCasEV2.1 induction. Three gRNAs targeting the positions −376, −300 and −150 were generated and tested with scRNA2.1 (Figure 1f). The p*SlDFR* activation assay was also interrogated with SlANT1, a Myb factor than naturally activates the *DFR* gene in tomato. As shown in Figure 1g, dCasEV2.1 activation rates matched those obtained with SlANT1 (× 400‐fold) when an appropriated gRNA (position −150) was used. dCasEV2.1 was also tested in *N. benthamiana* reporter lines carrying stably transformed pNos:Luc (Nos‐RL3, Nos‐RL5 and Nos‐RL6) or pDFR:Luc (DFR‐RL1) constructs. For each line, leaves were dCasEV2.1‐agroinfiltrated, with or without target‐specific gRNAs, and their reporter activation levels measured. As shown in Figure 1h, activation rates were in line with what was observed in transient experiments. Stably transformed pNos:Luc were induced 3‐ to 13‐fold reaching maximum levels similar to CaMV35S promoter; DFR‐RL1 line having lowest basal luminescence rates showed activation rates up to 90‐fold.

Next, we analysed the ability of dCasEV2.1 to activate transcription of endogenous *N. benthamiana* genes. For this purpose, we targeted the *N. benthamiana* homologue of DFR gene (*NbDFR*) and a transcription factor (*NbAN2*) involved in polyphenol biosynthesis, which can also trigger *NbDFR* expression. In these experiments, we followed a multiplexing strategy (Xie *et al*., 2015), targeting each gene with groups of three gRNAs expressed in multicistronic transcripts as depicted in Figure 1i. Results showed a strong 10 000‐fold activation of *NbDFR* with dCasEV2.1 loaded with six gRNAs combination, outperforming that obtained with endogenous Myb *NbAN2* (Figure 1j). Similarly, *NbAN2* reached maximum activation rates (>4000×) with a 6× multiplex gRNA combination (Figure 1l).

The powerful dCasEV2.1‐triggered induction observed in endogenous genes prompted us to investigate whether enhanced transcriptional activation had occurred at the expenses of specificity. To explore this, we performed RNAseq analysis of leaf samples treated with a dCasEV2.1 directed against *NbDFR* (Niben101Scf00305g05035) and *NbAN2* (Niben101Scf00156g02004) promoters, using the same 6× gRNAs combinations as described above. An off‐target analysis for all six *NbDFR* gRNAs identified only a single high‐score potential off‐target (Niben101Scf00606g02015), labelled as *NbDFR*(h), known to be a *NbDFR* homologous genes. *NbDFR*(h) promoter sequence had a full match with gRNA a (−88) and also showed two mismatches with gRNA f (−248). The differential gene expression analysis at 4 dpi is represented in Figure 1k, with the fold change between *NbDFR*‐induced and control samples represented in the *y*‐axis and the log counts per million (CPM) indicative of absolute expression levels plotted in the *x*‐axis. As it can be observed, upon dCasEV2.1/*NbDFR* activation, the leaf transcriptome remains virtually unchanged except for the *NbDFR* gene itself and its previously identified off‐target, *NbDFR*(h). Strikingly, *NbDFR* is induced from extremely low expression levels to the top 50 highly expressed mRNA in the transcriptome. No significant category enrichments of up‐regulated and down‐regulated genes were found in *NbDFR*‐activated sample (support information at https://doi.org/10.5281/zenodo.2559195). Similar results were observed when targeting *NbAN2* promoter. Off‐target analysis identified an *NbAN2* homologue [Niben101Scf00285g10004, *NbAN2*(h)], as potential secondary target, showing two full matches [gRNAs a (−103) and d (−145)] and three additional partial matches [gRNAs b (−175), c (−196) and e (−198)]. As in the previous example, the differential gene expression analysis plotted in Figure 1m revealed that *NbAN2* was the strongest activated gene in the transcriptome, followed by *NbAN2*(h) as anticipated in off‐target analysis. Contrary to what was observed for *NbDFR*,* NbAN2* activation was accompanied by more significant changes in the transcriptome, as expected for a TF, although most of the changes observed had fold changes far below those observed in the targeted genes. Interestingly, *NbDFR* was also detected among significantly activated genes, although with much lower log CPM and fold change values that in direct activation.

In sum, dCasEV2.1 is an optimized PTA shown to act efficiently at two different levels: with heavily connected master regulators and with final actuator enzymes. This new tool combines maximum potency with high specificity and genome‐proven orthogonality, therefore opening the way to powerful applications in plant SynBio.

## Conflict of interest

The authors declare no conflicts of interest.
